# Effect of liraglutide on depressive symptoms in overweight or obese patients with type 2 diabetes: protocol for a pilot randomized controlled trial

**DOI:** 10.3389/fendo.2025.1629157

**Published:** 2026-01-21

**Authors:** Wei Fang, Zinan Li, Jie Xu, Jing Zhao, Hongxia Zhu, Huanping Wang

**Affiliations:** Department of Endocrinology, Chengdu Shuangliu Hospital of Traditional Chinese Medicine, Chengdu, China

**Keywords:** depression, glucagon-like peptide-1 receptor agonists, liraglutide, obesity, type 2 diabetes

## Abstract

**Introduction:**

Patients with concurrent obesity, type 2 diabetes, and depression experience high disease severity and prevalence. This triad of conditions compromises quality of life and treatment adherence, further exacerbating disease progression. Therapeutic strategies for such patients must address both glycemic control and psychological well-being. Liraglutide, a glucagon-like peptide-1 receptor agonist (GLP-1RA), offers benefits beyond glucose-lowering and weight reduction, with emerging evidence suggesting it may also alleviate depressive symptoms. Therefore, liraglutide represents a promising intervention for managing depression in patients with obesity and diabetes.

**Objectives:**

This study aims to assess the therapeutic efficacy of liraglutide in overweight or obese patients with type 2 diabetes and comorbid depression, with a specific focus on its antidepressant effects.

**Methods:**

This is a randomized, double-blind, placebo-controlled pilot trial. Sixty eligible participants will be randomly assigned (1:1) to receive either liraglutide (initiated at 0.6 mg/day, titrated weekly to a maximum of 1.8 mg/day) or a matched placebo, as an adjunct to standard care for 12 weeks. The primary endpoints include blood glucose levels, glycated hemoglobin, body mass index, Hamilton Depression Rating Scale score, and metrics derived from resting-state functional magnetic resonance imaging (resting-state fMRI). Secondary endpoints will assess changes in inflammatory biomarkers (tumor necrosis factor-α, interleukin-6), oxidative stress indicators (superoxide dismutase, malondialdehyde), homeostasis model assessment of insulin resistance, insulin sensitivity index, and homeostasis model assessment of β-cell function.

**Conclusions:**

This trial will provide preliminary data on the effects of liraglutide on depressive symptoms in overweight/obese patients with type 2 diabetes. The findings are expected to provide a basis and reference for subsequent large-scale clinical research.

## Introduction

### Background and rationale {6a}

Metabolic disorders, particularly obesity and diabetes, have become increasingly prevalent; recent epidemiological studies indicate that the global population affected by obesity has exceeded 2 billion (1), while the prevalence of diabetes is also on the rise (2).Today, type 2 diabetes(T2DM) and obesity are widely recognized as “co-occurring diseases” resulting from the interplay of genetic and environmental factors. Furthermore, overweight and obesity play a critical role in the development and progression of T2DM (3, 4). Notably, both obesity and T2DM are not only significant risk factors for disability and mortality, but also have a profound impact on emotional well-being (5).Research shows that metabolic diseases and mental disorders (especially depression) have a bidirectional relationship (6, 7).The risk of depression in patients with T2DM is 2–3 times higher than that in the general population, and the risk of developing T2DM in patients with depression is also significantly increased (8). A survey study found that the rate of depressive symptoms in patients with T2DM was as high as 68.2% (9), and an overweight/obese status may further increase the risk of emotional disorders in this group (10). Obesity and type 2 diabetes are considered physiological and psychosomatic diseases closely related to psychological factors (11).

Since metabolic diseases, such as obesity and T2DM, frequently co-occur with mood disorders like anxiety and depression, some scholars have introduced the concept of the metabolic-affective syndrome and suggested that there may be common underlying mechanisms for obesity, T2DM, and depression (11).In-depth mechanistic investigations have revealed that the pathological network underlying metabolic affective syndrome encompasses multiple pathways, such as neuroendocrine dysregulation, chronic low-grade inflammation, and insulin resistance, as well as other factors (12, 13).Current clinical management demonstrates persistent limitations in the efficacy of conventional antidepressant therapies and glycemic control protocols for patients with T2DM and comorbid depression (14), highlighting the lack of targeted therapeutic strategies for metabolic affective syndrome.

Glucagon-like peptide-1 (GLP-1), a hormone secreted by intestinal L-cells, exhibits glucose concentration-dependent hypoglycemic effects. Its receptor agonists (GLP-1RAs) mimic the physiological actions of GLP-1 and have been widely used as glucose-lowering agents in the clinical management of T2DM, particularly in overweight/obese patients (15, 16). GLP-1RAs and their analogs can cross the blood-brain barrier and directly influence the physiological functions of the human brain (17). Basic studies reveal that GLP-1RAs exert neuroprotective effects through multiple pathways such as inhibiting neuroinflammation, promoting neurogenesis, and restoring neuronal insulin signaling (18, 19). Preclinical studies have shown that liraglutide treatment significantly improves cognitive function in patients with depression and bipolar disorder (20). However, current clinical evidence remains controversial. Although recent meta-analyses support the efficacy of GLP-1RAs in improving depression scale scores (21), some studies suggest potential risks of inducing mood fluctuations or depressive symptoms (22).This inconsistency in evidence reflects the complexity of disease mechanisms and highlights the importance of conducting rigorous clinical studies.

To this end, the project team designed a randomized, controlled, double-blind clinical trial: Effect of Liraglutide on Depressive Symptoms in Overweight or Obese Patients with Type 2 Diabetes: Protocol for a Pilot Randomized Controlled Trial. The findings of this study will offer supportive evidence for liraglutide use in overweight or obese patients with type 2 diabetes mellitus comorbid with depression, and lay the groundwork for a definitive large-scale clinical study.

### Objectives {7}

This study aims to evaluate the clinical efficacy of daily liraglutide treatment in obese and overweight patients with comorbid T2DM and depression as well as its impact on depressive symptoms. This study adopts a multidimensional assessment system: 1) metabolic parameters, with focused monitoring of glycemic control, body weight changes, and insulin sensitivity improvements; 2) mental health evaluation, with dynamic assessments using standardized depression scales combined with resting-state functional magnetic resonance imaging (rs-fMRI) to investigate brain functional alterations; and 3) pathogenesis exploration, with assessment of the changes in inflammatory and oxidative stress markers. The results of this study are expected to demonstrate that liraglutide can improve metabolic parameters and depression scores in overweight or obese patients with T2DM comorbid with depression, providing evidence for GLP-1RA intervention in “metabolic-affective syndrome. Furthermore, through systematic analysis of the aforementioned biomarkers, this study aims to preliminarily elucidate the potential mechanism underlying liraglutide’s benefits—specifically, whether it exerts concurrent positive effects on both metabolism and depressive symptoms via a synergistic pathway involving the reduction of systemic low-grade inflammation, alleviation of oxidative stress, and improvement of insulin signaling.

### Trial design {8}

**Figure 1:**
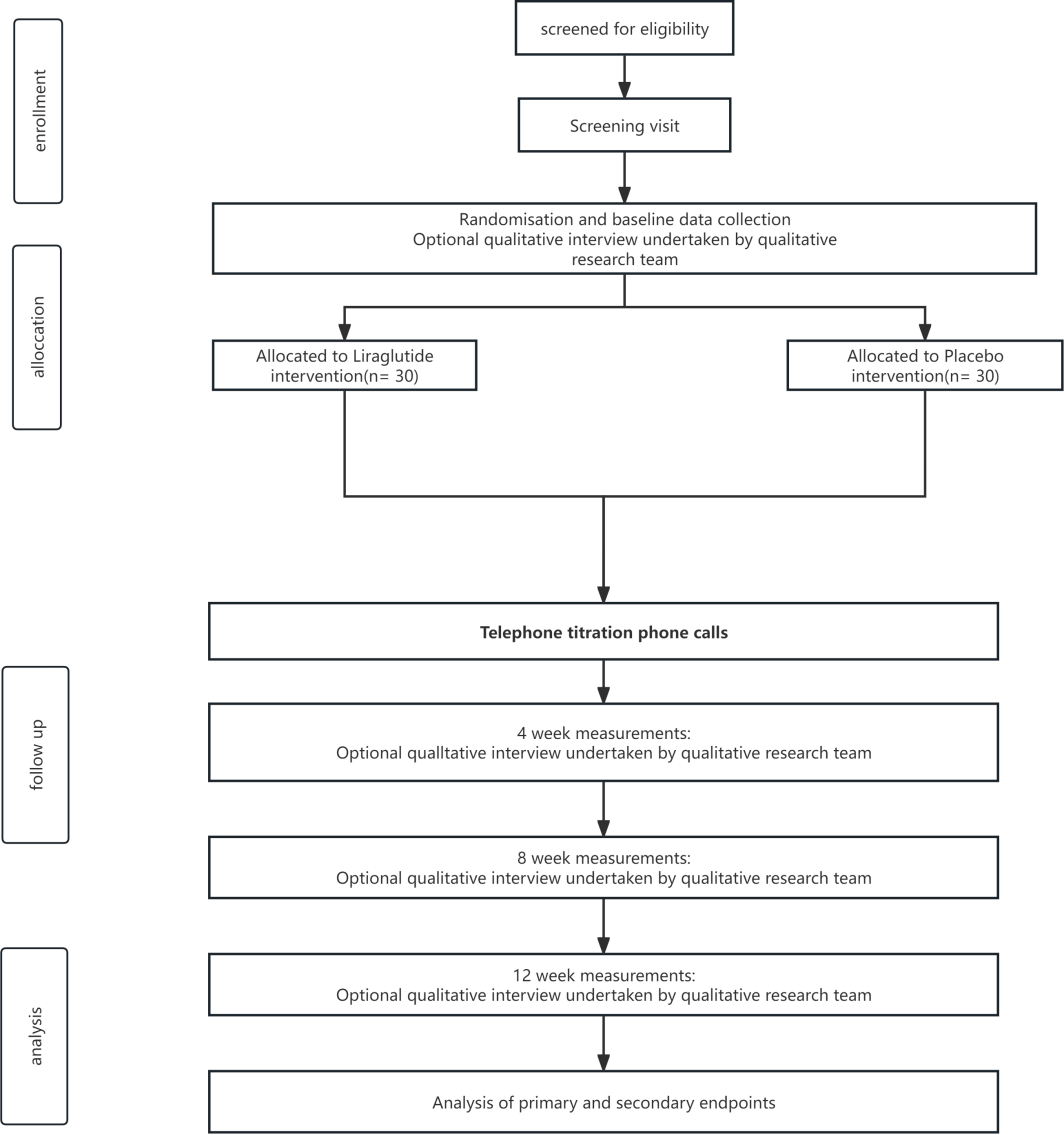
Flow diagram of the recruitment process, group allocation, and participation in the two interventions.

This randomized, controlled, double-blind pilot trial will be conducted in Chengdu, China. Eligible participants will be randomly allocated to two parallel groups (1:1 ratio). All participants will retain the right to withdraw at any stage, consistent with the Standard Protocol Items: Recommendations for Interventional Trials (SPIRIT) guidelines (23). **Figure 1** presents a flowchart of the study.

## Methods: participants, interventions, and outcomes

### Study setting {9}

The investigation will be conducted at a prestigious third-grade Class A medical institution in Shuangliu District, Chengdu. This hospital is recognized as the largest and prominent center for traditional Chinese medicine in the region, serving nearly two million residents. Equipped with state-of-the-art diagnostic and treatment facilities, patients who are enrolled in the study will receive their follow-up care, undergo necessary medical examinations, receive drug interventions, and fill out the required questionnaires within the hospital’s endocrinology department.

### Eligibility criteria {10}

The diagnostic and screening process for depression in this study will be conducted in two stages. Initial screening based on the Hamilton Depression Rating Scale (HAMD-24) will be performed by trained research staff. Subsequently, a definitive diagnosis of depressive disorder will be confirmed by a board-certified psychiatrist with over ten years of clinical experience, using the Mini-International Neuropsychiatric Interview (MINI) aligned with ICD-11 criteria. All raters administering the HAMD will undergo standardized training, and inter-rater reliability will be assessed prior to the main trial using an independent sample; the results (Intraclass Correlation Coefficient) will be reported to ensure consistency. Endocrinologists will be primarily responsible for assessing metabolic inclusion criteria and overall medical safety. The screening protocol will involve an array of assessments to ensure comprehensive evaluation of each participant. These include baseline demographic and anthropometric measurements (height, weight, and body mass index), laboratory investigations (fasting blood glucose levels, glycated hemoglobin levels, complete blood count, and routine biochemical profiles), and psychological evaluations using standardized tools such as the Hamilton Depression Rating Scale. In addition, an exhaustive review of each participant’s medical history will be conducted, and eligible individuals will be provided with a detailed explanation of the aims and objectives of the study. If they indicate a desire to participate, a personal interview will be arranged to address any questions and verify that they meet all specified inclusion and exclusion criteria. In this interview, a member of the research team from the trial steering committee will present the study details to potential participants and obtain written informed consent. Screening will continue until the target number of participants is achieved. **Table 1** lists the inclusion criteria and **Table 2** lists the exclusion criteria.

**Table 1:** Inclusion criteria.

Inclusion of criteria entries	
(1)	Age ≥18 years
(2)	Meeting the diagnostic criteria for Type 2 diabetes (24), with poor glycemic control after lifestyle intervention with 7.0% ≤ glycated hemoglobin ≤ 9.0% and fasting blood glucose ≤ 11.1 mmol/L
(3)	Body mass index greater than 24.0 kg/m²
(4)	Meeting the diagnostic criteria for mild depression without psychotic symptoms (25)
(5)	A score on the Hamilton Depression Rating Scale (HAMD, 24-item version) of ≥8 and ≤35 (26)
(6)	No use of any antidepressant or psychotropic medications in the past two weeks
(7)	The patient’s condition was stable and met the requirements of follow-up evaluation
(8)	Voluntary participation in the trial and signing of an informed consent form

**Table 2:** Exclusion criteria.

Exclusion of criteria entries	
(1)	In cases of combined severe infections or significant functional disorders of crucial organs, such as: ALT or AST ≥ 2.5 times the upper limit of the normal value, or total bilirubin ≥ 2 times the upper limit of the normal value; Impaired renal function: glomerular filtration rate (eGFR, CKD-EPI formula) < 60 mL/min/1.73m2, calcitonin ≥ 50 ng/L; Serum amylase or lipase ≥ 3 times the upper limit of the normal value; Severe arrhythmias, heart failure, etc., are not suitable for participating in the trial
(2)	Those with other severe mental symptoms, such as combined organic mental disorders, alcohol and drug dependence, schizophrenia, bipolar disorder, etc.; Patients with a suicide risk
(3)	Subjects with persistent gastrointestinal symptoms such as nausea and vomiting within 6 months prior to enrollment, a history of gastroparesis, uncontrolled gastroesophageal reflux disease, gastric ulcer or pancreatitis; Currently presenting with diabetic ketoacidosis or diabetic hyperosmolar state
(4)	Subjects with a known history of drug abuse or long-term heavy alcohol consumption, causing physical and mental dependence, with an average daily intake of pure alcohol exceeding 25g (equivalent to 750 mL of beer, 250 mL of wine, or 50g of spirits) within 3 months prior to screening
(5)	Subjects currently participating in other drug clinical trials or having used DPP-4 and GLP-1 related preparations within 3 months.
(6)	Subjects currently suffering from perimenopausal syndrome; Pregnant or lactating women; or those allergic to the trial drug
(7)	Subjects with a previous history of medullary thyroid carcinoma or a family history of type 2 multiple endocrine neoplasia syndrome

### Who will take informed consent? {26a}

Before potential participants are enrolled in the clinical trial, the responsible physician will provide a detailed explanation of all aspects of the study. This information included the nature of the interventions, potential risks related to participation, anticipated benefits, and other relevant details. If participants choose to participate after fully understanding the study, its risks, and potential benefits, they or their legally authorized representatives will sign a written informed consent document to confirm their voluntary agreement to participate.

### Additional consent provisions for collection and use of participant data and biological specimens {26b}

The informed consent process includes participants’ agreement to the use of their personal data as part of the research. No additional analyses will be performed on the biological samples.

## Interventions

### Explanation for the choice of comparators {6b}

The trial is designed as a placebo-controlled study with a 1:1 randomization ratio between liraglutide and placebo. Patients in both study arms will receive a structured dietary management plan and guidance on diabetes-specific physical activity. They will be required to adhere consistently to the prescribed diet and exercise regimen for 12 weeks.

### Intervention description {11a}

#### Randomization and intervention

Upon providing written informed consent, participants will be randomly allocated in a 1:1 ratio to either the experimental or control group. The experimental group will receive active treatment with liraglutide injections, whereas the control group will receive matching placebo injections. Treatment protocols will be determined by two endocrinologists and a psychiatrist involved in screening. Both groups will receive identical standard care, including diabetes-specific dietary plans and exercise guidance throughout the 12-week study period. Outcomes will be assessed at four time points: baseline and weeks 4, 8, and 12 (dual assessment at week 12 for the interim and final evaluations).

#### Experimental group protocol

Participants will initiate liraglutide at 0.6 mg/day for two weeks, with weekly 0.6 mg dose escalation to reach the target dose of 1.8 mg by week 2. Following a 2-week titration phase, the dose will be maintained at 1.8 mg for the remaining 10 weeks. Subcutaneous administration via a prefilled pen will occur daily at self-selected consistent times at preferred injection sites (the upper arm, thigh, or abdomen). For participants intolerant of 1.8 mg, the dose will be temporarily reduced to 1.2 mg, followed by two re-escalation attempts at 2-week intervals. Those who fail both attempts will continue on 1.2 mg.

#### Control group protocol

Placebo recipients will follow identical dosing schedules to the experimental group, including matched titration phases and injection procedures. Dose adjustments will be synchronized between the groups to maintain blinding integrity.

### Criteria for discontinuing or modifying allocated interventions {11b}

Individuals will be removed from the study under specific conditions: 1) participants who consistently violate the study protocol, fail to comply with the medication regimen, or for whom treatment efficacy cannot be determined due to incomplete data; 2) participants who manifest hypersensitivity reactions or are intolerant to the study medication, with special attention to those exhibiting exacerbation of depressive symptoms or suicidal ideation, requiring immediate cessation of study participation and the initiation of psychological intervention; 3) participants who opt to withdraw from the clinical trial and cease treatment; 4) participants in whom the average fasting blood glucose level reaches or exceeds 11.1 mmol/L during follow-up and this threshold is not reduced after a one-week reassessment; and 5) participants exhibiting emergence of acute or chronic pancreatitis, thyroid abnormalities, or other endocrine disorders during the treatment period, or significant deviations in liver function or serum amylase levels beyond the normal range. For all instances where the participants is discontinued, the rationale must be documented, and the corresponding case report forms must be preserved for subsequent review.

### Strategies to improve adherence to interventions {11c}

Our study team will devise and implement regular communication strategies to enhance the adherence of participants to the research interventions. This strategy will involve weekly communication with participants via WeChat and telephone, which will not only help increase their engagement and enthusiasm for the study, but also will allow us to stay informed about their psychological well-being. Through these communication channels, the research team will closely monitor and identify any potential side effects or adverse events experienced by participants, thereby ensuring the safety and efficacy of the study.

### Relevant concomitant care permitted or prohibited during the trial {11d}

Throughout the study, foundational treatment will be emphasized for patients with diabetes. Participants are permitted to continue their pre-existing, stable (≥4 weeks of use prior to enrollment) conventional glucose-lowering medications—including metformin, sulfonylureas, sodium-glucose cotransporter-2 inhibitors, insulin and its analogues—in addition to the investigational product (liraglutide or placebo), in order to comply with ethical standards and maintain glycemic stability, while the initiation or switch to dipeptidyl peptidase-4 inhibitors, other GLP-1 receptor agonists, and related preparations is strictly prohibited. To ensure comparability of background therapy between groups, we will implement stratified randomization based on baseline insulin use and will meticulously record and compare all baseline glucose-lowering medications across groups prior to analysis to confirm randomization balance.

For the basic treatment protocol, the study will adhere to the recommendations outlined in the Chinese Diabetes Prevention and Treatment Guidelines (2020 edition).

### Provisions for post-trial care {30}

After the trial concludes, the participants will undergo regular mental health screenings to monitor for any long-term side effects related to depression.

### Outcomes {12}

Observational indicators include two types: efficacy and safety indicators.

### Efficacy indicators

#### Primary outcome measures

1) Efficacy indicators include fasting plasma glucose, 2-hour postprandial glucose, body mass index (BMI), glycated hemoglobin (HbA1c), and lipid profiles (total cholesterol, triglycerides, low-density lipoprotein cholesterol, and high-density lipoprotein cholesterol).
2) Depression scale scores, derived from the Hamilton Depression Scale (HAMD), will be used to assess the severity of depressive symptoms. This validated clinical tool provides quantifiable measurements of both condition severity and treatment response in depression management (27).
3) Resting-state functional magnetic resonance imaging parameters comprise three key metrics: the amplitude of low-frequency fluctuations (ALFF), regional homogeneity (ReHo), and functional connectivity (FC). These biomarkers quantitatively reflect spontaneous neural activity patterns and dynamic connectivity alterations across brain networks. They are widely employed in neuropsychiatric research, particularly in depression studies (28).

### MRI acquisition protocol

Neuroimaging data will be acquired using a Siemens 3T Trio MRI system (Erlangen, Germany) equipped with an 8-channel phased-array head coil. Prior to scanning, the participants will receive standardized instructions to maintain their eyes closed, relax without focused thinking, and minimize head motion during acquisition. For obtaining sagittal T1-weighted imaging structural images, a three-dimensional magnetization-prepared rapid gradient echo sequence will be employed, with the following specific parameters: TR 1900 ms, TE 2.52 ms, TI 900 ms, a flip angle of 9°, 160 slices, a voxel size of 1 mm × 1 mm × 1 mm, a field of view of 250 mm × 250 mm, and a matrix resolution of 256 × 256. Resting-state brain functional images will be acquired using a planar gradient echo sequence with the following specific parameters: TR 2000 ms, echo time 30 ms, flip angle 90°, 33 slices, slice thickness 4 mm. field of view 240 mm × 240 mm, matrix 64 × 64, and total acquisition 200 time points. Furthermore, conventional T2-weighted imaging and fluid-attenuated inversion recovery sequences will be used to exclude potential white matter degeneration or lacunar infarct foci.

### Preprocessing and index calculation of rs-fMRI data

All preprocessing will be performed using the DPABI v6.0 toolbox in MATLAB software (29). To achieve data balance, the first 10 time points of each participant’s data will be excluded. Subsequently, the following preprocessing steps will be implemented: (1) temporal layer correction; (2) head motion correction, with exclusion of data if the participant’s head translation exceeds 2 mm or rotation exceeds 2°; (3) covariate regression (including Friston 24 head motion parameters, global brain signal, white matter signal, and cerebrospinal fluid signal); (4) spatial normalization (resampling voxel size to 3 mm × 3 mm × 3 mm); (5) spatial smoothing (Gaussian kernel full width at half maximum of 6 mm); (6) removal of linear drift; and (7) band-pass filtering (bandwidth of 0.01–0.10 Hz).

Resting-state brain functional indices will be derived using DPABI v6.0. The amplitude of low-frequency fluctuations will be computed after the aforementioned preprocessing stages, focusing on ALFF values within the frequency range of 0.01–0.08 Hz. The regional homogeneity values reflect Kendall’s coefficient of concordance, encompassing a voxel and its 26 neighboring voxels. Spatial smoothing will not be applied during preprocessing; however, Gaussian smoothing will be conducted after computation using a smoothing kernel diameter of 6 mm.

Given the asymmetry of the human brain, a symmetric template in the standard space will be constructed from the T1-weighted structural images of all participants. This template will subsequently be utilized to re-standardize the functional images. Following spatial smoothing, removal of linear drift, and filtering, the Pearson correlation coefficient between symmetric voxels across both cerebral hemispheres will be calculated as the mean volume homogeneity correlation value. These indices will undergo Fisher-z transformation to generate z-values, which will then be used for further statistical analysis.

### Secondary indicators

Secondary indicators include changes in inflammatory factors [tumor necrosis factor-α (TNF-α), interleukin-6 (IL-6)]; oxidative stress markers [superoxide dismutase (SOD), malondialdehyde (MDA)]; homeostatic model assessment of insulin resistance; insulin sensitivity index; and homeostatic model assessment of β-cell function.

### Safety indicators

Safety indicators include adverse drug reactions, hypoglycemic reactions, vital signs, electrocardiograms, and hematological and biochemical indicators, including routine blood tests and liver and kidney functions. During follow-up, close monitoring of changes in the patients’ mood and vigilance regarding the risk of suicide is essential.

### Participant timeline {13}

Outcome assessments will be performed at baseline, followed by three subsequent visits. At baseline assessment, we will gather data on patient demographics, including age, gender, educational level, employment status, marital status, and comorbidities. Additionally, we will document the date of initial diagnosis, symptom duration, and details of prior medications and treatment regimens. The participants are required to revisit the hospital at 4, 8, and 12 weeks after medication initiation for a series of evaluations. These include height and weight measurements, completion of pertinent laboratory tests, and the administration of depression and cognitive function scales. These assessments aim to provide a comprehensive evaluation of treatment efficacy. The detailed enrollment process and comprehensive schedule of the evaluation results are presented in **Table 3**.

**Table 3:** Schedule of outcome measures and trial-related activities.

Items	Enrollment day	4 weeks	8weeks	12 weeks
Inclusion screening				
Sign informed consent	*√*			
Record general information	*√*			
Gender	*√*			
Age	*√*			
Body weight	*√*	*√*	*√*	*√*
height	*√*	*√*	*√*	*√*
Waist circumference	*√*	*√*	*√*	*√*
Hip circumference	*√*	*√*	*√*	*√*
Medical history and treatment history	*√*			
Combined disease and medication records	*√*			
life history	*√*			
Safety				
blood routine examination	*√*			*√*
routine urine test	*√*			*√*
stool routine examination	*√*			*√*
liver function	*√*			*√*
renal function	*√*			*√*
Electrocardiograph	*√*			*√*
Adverse events and complications		*√*	*√*	*√*
Efficacy index				
Hamilton Depression Scale 24 scores	*√*			*√*
Blood glucose (fasting and 2 hours after meals)	*√*			*√*
glycosylated hemoglobin	*√*			*√*
blood lipids	*√*			*√*
tumor necrosis factor-α, Interleukin-6	*√*			*√*
super Oxide Dismutase,malondialdehyde	*√*			*√*
HOMA Insulin resistance Index	*√*			*√*
Insulin sensitivity Index	*√*			*√*
HOMA Islet beta Cell Function Index	*√*			*√*
resting-state functional magnetic resonance imaging	*√*			*√*

### Sample size {14}

Determining the appropriate sample size is critical when devising a clinical trial, including a pilot study. These preliminary trials are designed to assess the feasibility and address pragmatic considerations for subsequent full-scale trials. Consequently, the sample size for these pilot studies is calibrated to provide estimates of study parameters with an acceptable level of precision rather than being predicated on traditional hypothesis testing (30). Sim et al. recommended a minimum of 50 participants (25 per group) to achieve the pilot trial feasibility goal (31). In previous clinical studies on mental disorders, 10% of the liraglutide group and 2% of the placebo group withdrew from the trial after 16 weeks (32). However, in a study of liraglutide in overweight/obese patients with type 2 diabetes, only two patients in the liraglutide group (198 cases) and four patients in the placebo group (198 cases) withdrew from the trial after 56 weeks (33). Based on data from other pilot trials of liraglutide (34), we assumed that the conservative dropout rate at 3 months would be between 10% and 15%; therefore, we would need to recruit approximately 60 participants (every 30) to provide a reliable estimate.

### Recruitment {15}

Eligible participants will be identified by reviewing the outpatient and inpatient records from the endocrinology department. For patients who fulfill the inclusion criteria, an invitation to participate and the trial information will be sent to their email address. Following the receipt of the written invitation, we will make a telephone call within one to two weeks to ascertain their interest in the study. Patients who desire to participate will be provided with a comprehensive study protocol and an outline of the process to enhance their understanding of the trial scope and procedures.

### Assignment of interventions: allocation

#### Sequence generation {16a}

After obtaining informed consent and post-baseline assessments, researchers will be alerted to initiate the group allocation process. Participants will be randomly assigned to one of the two groups at a 1:1 ratio using a system of generated random numbers. These numbers will be stored in opaque sealed envelopes, from which each participant will randomly selected one, thereby determining their allocation to either the treatment or control group. Patients will be evenly distributed between two interventions: once-daily administration of placebo in a blinded manner and once-daily liraglutide in a blinded manner. The hospital’s central pharmacy will be responsible for dispensing both placebo and the active drug. To maintain the integrity of the double-blind protocol, participants, researchers, and study monitors will not be privy to treatment assignments within the placebo and liraglutide groups at any time.

#### Concealment mechanism {16b}

Liraglutide and its placebo will be manufactured to be identical in appearance to ensure that the participants are blinded to the assigned medication. Sealed envelopes, each corresponding to a participant and containing details of the randomization sequence, will be stored securely to ensure that they are not accessible to the researchers at any time, thus preserving the integrity of the double-blind study design.

#### Implementation {16c}

The randomization procedure will be implemented using a web-based randomization system provided by the Chinese Clinical Trial Registry (http://www.medresman.org.cn/login.aspx).

### Assignment of interventions: blinding

#### Who will be blinded {17a}

A triple-blind design will be rigorously maintained throughout the trial period, encompassing all participants, healthcare personnel, and investigative staff from initial enrollment through the terminal follow-up assessment. Systematic disclosure of treatment allocations shall be deferred until study completion for all enrollees and conclusive verification of analytical results has been achieved.

#### Procedure for unblinding if needed {17b}

In accordance with the clinical research protocols, trial participants are required to immediately notify the principal investigator or research team upon experiencing adverse events. For serious adverse events, the principal investigator must conduct a causality assessment immediately after notification to evaluate the potential association with the investigational product. When deemed medically necessary, emergency unblinding procedures may be initiated by the investigator by accessing the confidential randomization code system maintained by the clinical trial pharmacy.

### Data collection and management

#### Plans for assessment and collection of outcomes {18a}

All the study-related data of the enrolled patients will be securely recorded using an electronic storage system. Patient data will be entered into an Excel database to ensure accuracy and confidentiality will be strictly maintained, with no disclosure of this information to researchers outside the study team until the conclusion of the study. Inspectors will conduct regular audits to review and correct entries, thereby ensuring the highest standards of data quality. Before the study begins, uniform standard operating procedures will be developed and comprehensive training will be delivered to the research staff, physicians, and nursing personnel at each participating center.

#### Plans to promote participant retention and complete follow-up {18b}

During the preliminary phase of the investigation, implementing weekly telephone follow-ups with individual participants will be used to increase retention rates. This structured engagement strategy serves a dual purpose: it enables investigators to obtain timely feedback regarding participants’ needs, while simultaneously fostering increased study commitment and mutual trust. The research protocol includes establishing multiple communication channels (e.g., phone, WeChat, email) with explicit instructions for emergency contact and ongoing support availability. These protocol enhancements are designed to ensure sustained participant comfort throughout the research lifecycle, thereby optimizing both intervention efficacy and overall satisfaction metrics.

### Data management {19}

Experienced research assistants will manage the collection and documentation of clinical data. Clinical examination results will be carefully documented in case report forms and entered into an electronic format. To ensure data entry accuracy, a dedicated technician will conduct consistency verification. All collected information will be stored securely in a password-protected digital system to maintain confidentiality. The study must comply with Good Clinical Practice guidelines, which emphasize safeguarding participants’ rights and well-being while ensuring data integrity and traceability for source verification. Participants retain the right to withdraw from the study at any time without justification, and this choice will not impact their future medical treatment or legal entitlements. After the completion of the study, participant records will be maintained for 3 years to enable future review and analysis.

### Confidentiality {27}

All personally identifiable records will be securely stored in a separate study database, and sharing will be strictly confined to the research context. Access to intervention-related data will be granted exclusively to authorized healthcare practitioners and principal investigators involved in the study.

#### Plans for collection, laboratory evaluation, and storage of biological specimens for genetic or molecular analysis in this trial/future use {33}

Upon blood collection, the specimens will undergo centrifugation and be promptly preserved at -80°C in laboratory freezers at the Chengdu Shuangliu District Traditional Chinese Medicine Hospital. All trial-related samples will be labeled with unique patient identification codes to maintain anonymity by excluding personally identifiable information. The participants retain the option of authorizing biological sample retention for potential future investigations. Following the conclusion of the study, de-identified specimens will be maintained in cryogenic storage for a 3-year period, after which they will be systematically incinerated in compliance with regional regulatory standards. No genomic profiling is currently intended for these biological materials.

### Statistical methods

#### Statistical methods for primary and secondary outcomes {20a}

The aim of this study is to compare the effectiveness of the two treatment regimens by assessing participants twice, once at baseline and again at the 3-month follow-up. To control for potential confounding variables, we will statistically adjust for participants’ age, gender, body mass index, and Hamilton Depression Rating Scale scores in accordance with the STRIVE criteria. Numbers and percentages will be used to express categorical variables and median and interquartile range will be used for continuous variables. Primary outcome measures (e.g., HAMD score, HbA1c, body weight) will be analyzed using Linear Mixed-Effects Models. The models will include treatment group, time, and the group-by-time interaction as fixed effects to test for between-group differences in treatment effect and its change over time. Subject-specific intercepts will be included as random effects. Analyses will be adjusted for pre-specified confounding variables, including age, sex, baseline BMI, and baseline HAMD score, which will be entered into the models as fixed-effect covariates. For missing data due to dropout, Multiple Imputation will be employed. Secondary outcomes and baseline characteristics will be described and compared as originally planned. All analyses will be performed using SPSS v.22.0 (SPSS Inc., Chicago, IL, USA) and the lme4 package in R. A p-value < 0.05 will be considered statistically significant, whereas a p-value <0.01 will be regarded as indicating a high degree of statistical significance.

The DPABI v6.0 toolkit will be used to perform two-sample t-tests on the whole-brain ALFF, ReHo, and VMHC maps of the two groups of participants. To correct for multiple comparisons across voxels, the statistical significance of the resulting maps will be assessed using Gaussian Random Field (GRF) theory, a method for family-wise error (FWE) rate correction. The threshold was set at a voxel-level significance of P < 0.001 and a cluster-level significance of P < 0.05.

##### Interim analyses {21b}

In the design of this study, no interim analyses are planned.

### Methods for additional analyses (e.g., subgroup analyses) {20b}

No additional analyses not described in Section 20a are planned.

### Methods in analysis to handle protocol non-adherence and any statistical methods to handle missing data {20c}

In this study, we will prioritize the utilization of data provided by participants who discontinue their involvement before the final follow-up. Any incomplete data as a result of participant withdrawal will be categorized as missing, and estimation or imputation methods will not be employed for these missing values.

### Plans to give access to the full protocol, participant-level data, and statistical code {31c}

Fully anonymized data and statistical codes will be made available in public databases after completion of the trial.

### Oversight and monitoring

#### Composition of the coordinating center and trial steering committee {5d}

The steering committee for this study consists of the principal investigator and two co-investigators who are responsible for overseeing the implementation of the study. Coinvestigators who are graduate students in the field of endocrinology will be involved in patient recruitment, obtaining informed consent, and follow-up. The principal investigator will independently generate a random allocation sequence and assign the participants to the two intervention groups. Additionally, the steering committee has the authority to review and approve necessary modifications to the study protocol.

#### Composition of the data monitoring committee, its role and reporting structure {21a}

Due to the simplicity of the study, a data monitoring committee is not required.

#### Adverse event reporting and harms {22}

Adverse events will be stringently monitored. The recruiting physician will oversee the management of these events. When a patient encounters an adverse event, assessors, therapists, or other personnel will provide immediate emergency care and notify the recruiting physician. The recruiting physician will then be tasked with reporting adverse events to the principal investigator and ethics committee. The principal investigator is responsible for thoroughly informing the ethics committee about the adverse events, including their contexts and relevant details. Additionally, the recruiting physician must ensure that all adverse events are meticulously recorded in the Clinical Report Form to maintain accurate documentation.

Furthermore, given the psychiatric risks in the study population, an independent Safety Monitoring Board (SMB) will be established to conduct periodic safety reviews. The SMB will consist of independent experts not involved in trial conduct. Its key responsibilities include: conducting regular meetings (at least every 3 months) to review all adverse events, with particular attention to psychiatric events (e.g., worsening depression, suicidal ideation) and assessing their relationship to the intervention; being empowered to provide independent recommendations to the principal investigator and ethics committee regarding trial continuation, modification, or termination based on safety evaluations; and performing expedited review and independent assessment of all serious adverse events.

#### Frequency and plans for auditing trial conduct {23}

The research team is expected to deliver a monthly progress update by the conclusion of each calendar month. This document will present a systematic evaluation of the ongoing investigative work while maintaining confidentiality regarding the empirical findings. The key components will include (1) achievement milestones relative to predetermined research goals, (2) status assessment comparing actual progress with projected timelines outlined in the original protocol, and (3) strategic recommendations for plan optimization based on emerging challenges or opportunities identified during implementation.

## Dissemination plans

The results of this study will be published in a peer-reviewed academic journal. Additionally, we intend to present the findings of this study at academic conferences at local, national, and international levels.

### Patient and public involvement

This study was conducted without direct participation from patient representatives or community members in its design phase. The research outcomes will be disseminated through established academic channels including peer-reviewed publications and scientific conferences, adhering to standard research dissemination protocols.

## Discussion

GLP-1RAs, known for their unique blood glucose-lowering and weight-reducing effects, have emerged as a new frontier in the treatment of diabetes and obesity, earning the distinction of being named one of Science magazine’s Breakthroughs of the Year 2023. These agents may have revolutionary applications in the treatment of mental health problems and neurodegenerative diseases (35).GLP-1RAs, beyond their established role in improving insulin resistance (36), may have therapeutic potential for mental health disorders, such as depression and bipolar disorder, as well as for neurodegenerative diseases associated with cognitive dysfunction, including Parkinson’s and Alzheimer’s diseases (37, 38). Recent animal studies suggested that semaglutide has the potential to combat depression and anxiety. Mice with depression associated with type 2 diabetes induced by a high-fat diet showed a significant alleviation of depressive and anxiety-like behaviors following treatment with semaglutide, along with enhanced cognitive function (39). In a clinical trial focusing on obese patients with type 2 diabetes or prediabetes, liraglutide mitigated early cognitive dysfunction in individuals with diabetes (40). However, several potential adverse effects associated with GLP-1RAs warrant careful consideration. One study suggested that individuals taking semaglutide may be at a disproportionately higher risk of suicidal thoughts, with a 45% increased risk compared with those taking other medications. Moreover, this risk was quadrupled in patients who concurrently used semaglutide and antidepressants. In contrast, the use of liraglutide alone was not associated with an increased risk of suicidal ideation (41). A retrospective cohort study published in Nature Medicine, which included the health records of over one million patients, suggested that GLP-1RAs are associated with a lower risk of suicidal ideation compared to other types of obesity and diabetes therapies; therefore, approaching these findings with caution and continuing to monitor for potential adverse effects are necessary (42). Some experts recommend caution when using GLP-1RAs in patients with a history or risk of mental illness, emphasizing the need for close monitoring of their emotional states. Therefore, this study will include patients with mild-to-moderate depressive symptoms to ensure close monitoring of mood changes throughout the trial and subsequent follow-ups.

Limitations of this study must be acknowledged: 1) The intervention duration of 12 weeks is relatively short, thus limiting the evaluation of the long-term efficacy (including whether improvements in metabolism and depressive symptoms can be sustained) and long-term safety profile of liraglutide; 2) The findings are derived from a single-center Chinese population and require validation in multicenter studies and non-Chinese populations. Considering these limitations, future definitive studies should significantly extend the observation period (e.g., to 6 months or longer) to comprehensively assess the durability of efficacy, long-term safety, and potential delayed effects.

## Data Availability

The original contributions presented in the study are included in the article/Supplementary Material. Further inquiries can be directed to the corresponding authors.
